# Associated factors related to participation in general health checkup and survey of the effect of low-dose radiation exposure on health of residents of Fukushima Prefecture after the Fukushima Daiichi nuclear power plant accident

**DOI:** 10.1016/j.pmedr.2020.101214

**Published:** 2020-10-02

**Authors:** Nobuaki Moriyama, Chihiro Nakayama, Masatsugu Orui, Yujiro Kuroda, Hajime Iwasa, Teruko Horiuchi, Takeo Nakayama, Minoru Sugita, Seiji Yasumura

**Affiliations:** a Department of Public Health, Fukushima Medical University School of Medicine, Fukushima, Japan; b Sendai City Mental Health and Welfare Center, Sendai, Japan; c Center for Integrated Science and Humanities, Fukushima Medical University, Fukushima, Japan; d National Institute of Advanced Industrial Science and Technology, Tsukuba, Japan; e Department of Health Informatics, School of Public Health, Kyoto University, Kyoto, Japan; f Toho University, Tokyo, Japan

**Keywords:** Fukushima Prefecture, Fukushima Daiichi nuclear power plant accident, Health checkup, Health literacy

## Abstract

The Fukushima Daiichi nuclear power plant accident caused radioactive contamination of the surrounding area. In addition to annual health checkups, a survey of the effects of low-dose radiation exposure on health among Fukushima Prefecture residents after the accident has been conducted. Despite health literacy (HL) being recognized as essential to health, its association with participation in these checkups and the survey remains unknown. We aimed to describe the HL status of the Fukushima Prefecture residents and to verify the hypothesis that HL is associated with participation in both checkup and survey. In a cross-sectional study, a questionnaire was sent to 2000 randomly sampled Fukushima Prefecture residents; data from 770 individuals were analyzed. Communicative and critical HL were measured using a 5-point scale. Factors associated with participation were examined using logistic regression. The survey’s valid response rate was 38.5%. The average HL score was 3.11 ± 0.81. HL was not associated with checkup or survey participation. Checkup participation was negatively associated with radiation anxiety (odds ratio 0.93, 95% confidence interval 0.86–0.99, p = 0.03). The HL of Fukushima Prefecture residents after the accident was relatively lower than that of the Japanese general population, which may be attributed to difference in educational background. The complexities involved in understanding the effects of radiation on the health of residents could explain why no association between HL and participation in a health checkup and survey was observed. Future studies with a longitudinal design should clarify causality between anxiety and checkup participation.

## Highlights

• Health literacy was lower in Fukushima Prefecture residents after the radiation accident.
• Educational background in participants may be associated with low health literacy.
• Health literacy wasn’t associated with checkup and radiation survey participation.
• Low radiation anxiety was positively associated with participation in checkup.
• A longitudinal study is needed to clarify if anxiety affects checkup participation.

## 1 Introduction

Health literacy (HL) is defined as people’s knowledge, motivation, and competency to access, understand, appraise, and apply health information to make judgments and decisions regarding healthcare, disease prevention, and health promotion to maintain or improve quality of life (Sørensen et al., 2012). HL is reported to be positively related to several health-promoting behaviors (Fernandez et al., 2016) and is recognized as essential to health. Low HL reportedly reduces the ability to act on and understand the advice of a health professional (Chew et al., 2004) and limits the ability to access and navigate the healthcare system (Kripalani et al., 2006).

On March 11, 2011, Japan was affected by the Great East Japan Earthquake. The subsequent tsunami damaged reactors of the Fukushima Daiichi Nuclear Power Plant. This accident resulted in the release of a large amount of radioactive materials into the air, causing widespread problems, not necessarily radiation-related physical health issues, but other physical, psychological, and social difficulties (Hasegawa et al., 2015). Specifically, the radiation accident led to health risks such as an increased incidence of obesity (Ohira et al., 2016). Furthermore, the disaster reportedly had negative effects on mental health, including increased suicide rates (Ohto, et al., 2015) and worsened subjective well-being (Tiefenbach and Kohlbacher, 2015). A previous study conducted in Minamisoma City and Soma City, located 10–50 km from the power plant, found that both evacuees and non-evacuees had a higher risk of diabetes mellitus in 2013 and 2014 compared with the baseline established between 2008 and 2010 (Nomura et al., 2016).

Under normal circumstances, annual health checkups are required by law in most Japanese communities and companies as part of efforts toward disease detection and prevention. Since participants of health checkups reportedly have a lower risk of mortality than non-participants (Hozawa et al., 2010), participation in health checkups has been encouraged in accordance with the concept of preventive medicine in Japan.

However, in the aftermath of the radiation accident, normal circumstances were disrupted. Consequently, Fukushima Prefecture launched the “Fukushima Health Management Survey (FHMS)” to monitor long-term health of its residents following the disaster and promote their future well-being (Yasumura et al., 2012). FHMS includes a thyroid ultrasound examination for all Fukushima children aged 18 years or younger, a comprehensive health check for all residents from the evacuation zones, an assessment of mental health and lifestyles of all residents from the evacuation zones, and a record of all pregnancies and births among women in the prefecture who were pregnant on March 11, 2011. Additionally, Fukushima Prefecture conducted measurements of internal radiation exposure in residents. Since the survey aims to support residents’ health, a high level of participation is desirable.

**Fig. 1:**
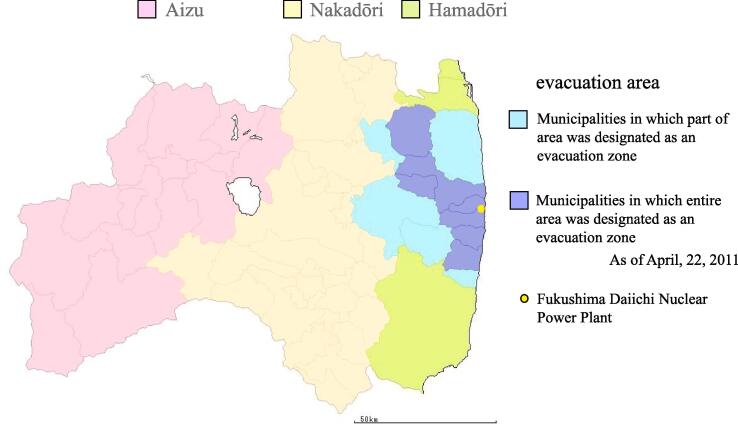
Area description of this study.

Several previous studies found that demographic factors (Power et al., 2009), socioeconomic status (Fukuda et al., 2005), as well as social support and network (Mitsuhashi et al., 2006) are associated with participation in health checkups. Moreover, general information about the checkup (Hislop et al., 2003) and the perceived benefits from it (Power et al., 2009) also promote participation. Although previous studies were conducted to develop a model for predicting HL among U.S. citizens (Martin et al., 2009) and to survey HL in Japanese office workers (Ishikawa et al., 2008), no study has elucidated the HL status and its relationship with Fukushima Prefecture residents’ participation in a health checkup and the survey after radiation accident. After the nuclear power plant accident, residents of the Fukushima Prefecture seemed to have been confounded by information obtained through the mass media (Yasumura, 2016). Thus, this study aimed to:(1) describe the HL status of residents of Fukushima Prefecture after the Fukushima Daiichi Nuclear Power Plant accident, (2) to explore factors associated with participation in the general health checkup and survey of the effect of low-dose radiation exposure on health, and (3) to verify a hypothesis that HL is associated with participation in both checkup and survey (Fig. 1).

## 2 Materials and methods

### 2.1 Subjects

This survey targeted 2,000 residents of the Fukushima Prefecture aged 20–79 years. We divided Fukushima Prefecture into 4 areas based on the general regional classification of Aizu, Nakadōri, and Hamadōri areas, as well as the evacuation area (restricted area, evacuation-prepared area, and deliberate evacuation area as determined on April 22, 2011) and selected 500 people from each area. The selection was based on a two-stage stratified random sampling method (Stage 1: selection of region; Stage 2: selection of individuals). Nakadōri and Hamadōri areas included local municipalities that were partially in the evacuation area; these were included in the evacuation area. The survey instrument was administrated as an anonymous, self-reporting postal questionnaire (Kuroda et al., 2018) between August 15 and October 17, 2016. A letter of request asking recipients to respond to the questionnaire voluntarily was attached. A returned questionnaire was considered as written informed consent to the objective of the study and voluntary participation. The present study was approved by the Ethics Committee of Fukushima Medical University (approval number: 2699).

### 2.2 Measured items

#### 2.2.1 Outcome

The following categories of health checkups and surveys were identified as objective variables:

(1) Regular health checkup conducted by a municipality or company,
(2) Health checkup other than that in category 1 (e.g. comprehensive health examination called “A Ningen Dock” in Japanese),
(3) Dosimetry of external exposure via a dosimeter (film badge), and
(4) Dosimetry of internal exposure via a whole-body counter
(5) Briefing session for thyroid ultrasound examination, and
(6) Lecture relevant to radiation organized by municipalities, or lecture on radiation and thyroid by medical doctors

Further, of these 6 categories, we identified that categories 1 and 2 were types of checkups (“checkups”), and categories 3 through 6 were types of surveys (“surveys”). Respondents who participated in at least 1 category within each type of test since the radiation accident occurred were categorized as participants; the remaining respondents were non-participants.

#### 2.2.2 Health literacy

To assess HL, we used the 5-point HL scale developed by Ishikawa et al. (2008) for use within the public. This scale was constructed to measure communicative HL (the ability to participate in everyday activities actively, to extract information, and to apply information to changing circumstances) and critical HL (the ability to analyze information critically and use this information to exert greater control over life events and situations). Communicative and critical HL is part of the HL model proposed by Nutbeam (2000). This scale determines whether respondents would be able to (1) collect health-related information from various sources, (2) extract their desired information, (3) understand and communicate the obtained information, (4) consider the credibility of the information, and (5) make decisions based on health-related issues.

Each item was rated on a 5-point scale, ranging from 1 (strongly disagree) to 5 (strongly agree). The individual HL status of each respondent was numerically assessed by obtaining the average scores of all 5 items.

#### 2.2.3 Covariates

Respondents were asked to report their age, gender, and their employment status (including those on leave). For area, the respondents were grouped into the evacuation area or “other” areas (Aizu, Nakadōri and Hamadōri areas). Respondents were also asked about self-rated health status; their educational background (junior high school, lower high school/junior college, vocational school/university, or graduate school); lifestyle habits including exercise, alcohol consumption, and smoking; anxiety about radiation; and experience of discrimination and prejudice. Responders’ self-rated health status was categorized into good (excellent, very good, as well as good) and not good (fair, unhealthy).

Regarding exercise habit, respondents were asked, “How many times do you play sports or exercise in a month?” These replies were categorized into no habit (never) and the presence of habit (others). Regarding alcohol consumption, respondents were asked, “Do you drink every day?” The replies were categorized into presence (yes) and absence (no and used to but quit). In addition, concerning smoking status, respondents were asked, “Do you smoke almost every day?” The replies were grouped into current smokers (yes) and non-smokers (no and used to but quit). Respondents’ anxiety about radiation and their experience of discrimination and prejudice were assessed by asking questions selected from a qualitative analysis of descriptions of worry, anxiety, and problems related to radiation exposure experienced by the evacuees in Fukushima Prefecture (Umeda et al., 2013). To assess respondents’ radiation anxiety, they were asked to respond to the following 4 statements: (1) I am concerned about getting a serious illness in the future due to the effects of radiation, (2) Every time I feel ill, I am afraid this is caused by radiation exposure, (3) I am concerned that radiation effects can be inherited by the next generation, such as children and grandchildren, and (4) I feel strong anxiety when I see news reports concerning the nuclear power plant accident.

To assess their experience of discrimination and prejudice, they were asked to respond to the following 3 statements: (1) I have had the experience of being discriminated against (or unfairly treated) because I lived in an area that was reported to have high levels of radiation, (2) I try not to tell others that I am a resident of that area as much as possible, and (3) I have experienced conflicts and trouble with my family members over radiation health effects. Respondents were asked to rate their answers to each question ranging from “strongly agree” (4 points) to “strongly disagree” (1 point). The sum of scores for each statement in the radiation anxiety category (4–16 points) and the discrimination and prejudice category (3–12 points) were assessed numerically; higher scores indicated a higher level of agreement (Kawakami, 2015).

### 2.3 Statistical analysis

To examine the degree of respondents HL, the score of communicative and critical HL (CCHL) was calculated. The second tertile of the average score for respondents was calculated to be 3.4; they were divided into 2 groups according to this score and those with a score higher than 3.4 were categorized into the high HL group; those with a score of 3.4 or less were categorized into the low HL group. Then, to examine the associated factor of HL in participants, univariate analysis was performed. In addition, to examine the association between HL and possible factors based on a conceptual causal model (Paasche-Orlow & Wolf, 2007); a chi-square test was performed to determine relationship of HL with age (20–44/45–64/65–79 years), employment status, and educational background (junior high school, lower high school/junior college, vocational school/university, or graduate school).

To explore the association between possible factors, including HL and participation in “checkups” and “surveys,” multiple logistic regression analysis was performed. HL was assessed using the CCHL score, gender, educational background, exercise habit, drinking habit, smoking habit, radiation anxiety, as well as discrimination and prejudice score as explanatory variables. Age (20–44/45–64/65–79 years; reference category: 20–44), district (evacuation zone/not evacuation zone), and employment status were treated as covariates. These variables were forced into the logistic regression model. All *p*-values were based on two-sided tests and *p*-values < 0.05 were considered statistically significant. All data were analyzed using IBM SPSS Statistics for Windows, version 21 (IBM Corporation, Armonk, NY, USA).

## 3 Results

In total, 916 people responded (response rate of 45.8%), of which incomplete or inadequate answers from 146 people were excluded, and we analyzed the remaining 770 responses (valid response rate of 38.5%). Percentage of valid responses out of whole responses was 84.1%. The number of valid respondents (valid response rates) by district was 183 (36.6%) in Hamadōri, 189 (37.8%) in Nakadōri, 230 (46.0%) in Aizu, and 168 (33.6%) in the evacuation zone.

The mean score for individual CCHL was 3.11 ± 0.81 (mean ± standard deviation). The mean for items 1 through 5 were 3.50 ± 1.04, 3.16 ± 0.98, 2.96 ± 1.00, 2.87 ± 0.92, and 3.08 ± 0.97, respectively. Comparison of age, educational background, and employment status between groups divided into HL level is shown in Table 1. Educational background was positively associated with HL (p < 0.01), whereas no association was found between HL and age as well as employment status (Table 1).

**Table 1:** Association between health literacy and age, educational background, and employment status.

		Health literacy			
		High n = 531 (%)	Low n = 239 (%)	Total n = 770 (%)	*p*-value^a^
Age (years)	20–44	132 (24.9)	62 (25.9)	194 (25.2)	0.52
	45–64	227 (42.7)	92 (38.5)	319 (41.4)	
	65–79	172 (32.4)	85 (35.6)	257 (33.4)	
Educational background	Junior high school or lower	19 (7.9)	80 (15.1)	99 (12.9)	**<0.01**
	High school	111 (46.4)	286 (53.9)	397 (51.6)	
	Junior college or vocational school	57 (23.8)	117 (22.0)	174 (22.6)	
	University or graduate school	52 (21.8)	48 (9.0)	100 (13.0)	
Employment status	Employed	320 (60.3)	155 (64.9)	475 (61.7)	0.26
	Unemployed	211 (39.7)	84 (35.1)	295 (38.3)	

^a^χ^2^ test or *t*-test.

Additionally, out of the 770 respondents, 528 (68.6%) participated in “checkups” and 226 (29.4%) participated in “surveys.” Univariate analysis shows that being male, low subjective health, residing in the evacuation zone at the time of the radiation accident, being employed, and low radiation anxiety were positively associated with “checkup” participation (p < 0.05; Table 2a). Further, residing in the evacuation zone and low radiation anxiety were associated with “survey” participation (p < 0.05; Table 2b).

**Table 2a:** Characteristics of responders according to participation in the general health checkup.

		Participation in general health checkup			
		Yes n = 528 (%)	No n = 242 (%)	Total n = 770 (%)	*p*-value^a^
Gender	Male	257 (48.7)	92 (38.0)	349 (45.3)	**<0.01**
	Female	271 (51.3)	150 (62.0)	421 (54.7)	
Subjective health^b^	Good	267 (50.6)	142 (58.7)	409 (53.1)	**0.04**
	Not good	261 (49.4)	100 (41.3)	361 (46.9)	
Age (years)	20–44	127 (24.1)	67 (27.7)	194 (25.2)	0.08
	45–64	233 (44.1)	86 (35.5)	319 (41.4)	
	65–79	168 (31.8)	89 (36.8)	257 (33.4)	
District	Non-evacuation zone	398 (75.4)	204 (84.3)	602 (78.2)	**<0.01**
	Evacuation zone	130 (24.6)	38 (15.7)	168 (21.8)	
Educational background	Junior high school or lower	61 (11.6)	38 (15.7)	99 (12.9)	0.23
	High school	269 (50.9)	128 (52.9)	397 (51.6)	
	Junior college or vocational school	124 (23.5)	50 (20.7)	174 (22.6)	
	University or graduate school	74 (14.0)	26 (10.7)	100 (13.0)	
Employment status	Employed	350 (66.3)	125 (51.7)	475 (61.7)	**<0.01**
	Unemployed	178 (33.7)	117 (48.3)	295 (38.3)	
Exercise habit^c^	Yes	287 (54.4)	121 (50.0)	408 (53.0)	0.26
	No	241 (45.6)	121 (50.0)	362 (47.0)	
Drinking habit^d^	Yes	167 (31.6)	63 (26.0)	230 (29.9)	0.12
	No	361 (68.4)	179 (74.0)	540 (70.1)	
Smoking habit^e^	Yes	109 (20.6)	54 (22.3)	163 (21.2)	0.40
	No	419 (79.4)	188 (77.7)	607 (78.8)	
Radiation anxiety	Mean ± SD	9.52 ± 2.86	10.02 ± 2.91	9.57 ± 2.89	**0.03**
Discrimination and prejudice	Mean ± SD	6.25 ± 2.11	6.14 ± 2.21	6.21 ± 2.14	0.50
Health literacy	High	169 (32.0)	70 (28.9)	239 (31.0)	0.39
	Low	359 (68.0)	172 (71.1)	531 (69.0)	

^a^χ^2^ test or *t*-test, ^b^Self-rated health status: Good (excellent, very good, good); Not good (fair, unhealthy), ^c^Exercise habit: “How many times do you play sports or exercise in a month?” Yes (1 to 3 times, 4 to 7 times, 8 to 15 times, more than 15 times); No (never), ^d^Drinking habit: “Do you drink every day?” Yes (yes); No (no, used to but quit), ^e^Smoking habit: “Do you smoke every day?” Yes (yes); No (no, used to but quit), Abbreviation: SD, standard deviation.

**Table 2b:** Characteristics of responders according to participation in the survey about radiation.

		Participation in radiation survey			
		Yes n = 226 (%)	No n = 544 (%)	Total n = 770 (%)	*p*-value^a^
Gender	Male	98 (43.4)	251 (46.1)	349 (45.3)	0.48
	Female	128 (56.6)	293 (53.9)	421 (54.7)	
Subjective health^b^	Good	109 (48.2)	252 (46.3)	361 (46.9)	0.63
	Not good	117 (51.8)	292 (53.7)	409 (53.1)	
Age (years)	20–44	63 (27.9)	131 (24.1)	194 (25.2)	0.50
	45–64	88 (38.9)	231 (42.5)	319 (41.4)	
	65–79	75 (33.2)	182 (33.5)	257 (33.4)	
District	Non-evacuation zone	143 (63.3)	459 (84.4)	602 (78.2)	**<0.01**
	Evacuation zone	83 (36.7)	85 (15.6)	168 (21.8)	
Educational background	Junior high school or lower	18 (8.0)	81 (14.9)	99 (12.9)	0.02
	High school	114 (50.4)	283 (52.0)	397 (51.6)	
	Junior college or vocational school	58 (25.7)	116 (11.8)	174 (22.6)	
	University or graduate school	36 (15.9)	64 (11.8)	100 (13.0)	
Employment status	Employed	87 (38.5)	208 (38.2)	295 (38.3)	0.95
	Unemployed	139 (61.5)	336 (61.8)	475 (61.7)	
Exercise habit^c^	Yes	96 (42.5)	266 (48.9)	362 (47.0)	0.10
	No	130 (57.5)	278 (51.1)	408 (53.0)	
Drinking habit^d^	Yes	68 (30.1)	162 (29.8)	230 (29.9)	0.93
	No	158 (69.9)	382 (70.2)	540 (70.1)	
Smoking habit^e^	Yes	41 (18.1)	122 (22.4)	163 (21.2)	0.19
	No	185 (81.9)	422 (77.6)	607 (78.8)	
Radiation anxiety	Mean ± SD	9.11 ± 2.78	9.93 ± 2.90	9.57 ± 2.89	**<0.01**
Discrimination and prejudice	Mean ± SD	6.14 ± 2.08	6.25 ± 2.17	6.21 ± 2.14	0.52
Health literacy	High	151 (66.8)	380 (69.9)	531 (69.0)	0.41
	Low	75 (33.2)	164 (30.1)	239 (31.0)	

^a^χ^2^ test or *t*-test, ^b^Self-rated health status: Good (excellent, very good, good); Not good (fair, unhealthy), ^c^Exercise habit: “How many times do you play sports or exercise in a month?” Yes (1 to 3 times, 4 to 7 times, 8 to 15 times, more than 15 times); No (never), ^d^Drinking habit: “Do you drink every day?” Yes (yes); No (no, used to but quit), ^e^Smoking habit: “Do you smoke every day?” Yes (yes); No (no, used to but quit), Abbreviation: SD, standard deviation.

Multivariate analysis found that “checkup” participation was positively associated with residing in the evacuation zone [OR: 2.17, 95% Confidence Interval (CI): 1.40–3.36] and being employed (OR: 2.11, 95% CI: 1.47–3.02), but was negatively associated with radiation anxiety (OR: 0.93, 95% CI: 0.86–0.99) (Table 3). Furthermore, residing in the evacuation zone at the time of the accident (OR: 3.37, 95% CI: 2.26–5.01), and a high level of education (OR: 1.37, 95% CI: 1.12–1.68) were positively associated with participation in “surveys” (Table 4).

**Table 3:** Factors associated with participation in the general health check-up.^a^

	Odds ratio	95% CI		*p*-value
		Lower limit	Upper limit	
HL: High (ref. Low)	0.94	0.65	1.34	0.72
Age (Ref. 20–44 years)	1.22	0.96	1.54	0.11
Gender: Female (ref. Male)	0.74	0.52	1.06	0.11
District: Evacuation zone (ref. Non-evacuation zone)	2.17	1.40	3.36	**<0.01**
Subjective health: Good (ref. Not good)	0.80	0.58	1.12	0.20
Exercise habit: Yes (ref. No)	1.14	0.82	1.58	0.42
Drinking habit: No (ref. Yes)	0.92	0.62	1.36	0.67
Smoking habit: No (ref. Yes)	1.37	0.90	2.08	0.14
Employment status: Employed (ref. Unemployed)	2.11	1.47	3.02	**<0.01**
Educational background (ref. Junior high school)	1.22	1.00	1.49	0.05
Radiation anxiety(For 1 point increase)	0.93	0.86	0.99	**0.03**
Discrimination and prejudice (For 1 point increase)	1.07	0.97	1.18	0.15

*p* < 0.05: statistically significant.

Abbreviations: CI, confidence interval; HL, health literacy; ref., reference.

Educational background: junior high school, lower high school/junior college, vocational school/university, graduate school; in ascending order.

^a^Logistic regression analysis.

**Table 4:** Factors associated with participation in the survey on the effect of low-dose radiation exposure on health.^a^

	Odds ratio	95% CI		*p*-value
		Lower limit	Upper limit	
HL: High (ref. Low)	0.99	0.69	1.43	0.96
Age (Ref. 20–44 years)	1.04	0.81	1.33	0.75
Gender: Female (ref. Male)	1.18	0.81	1.71	0.39
District: Evacuation zone (ref. Non-evacuation zone)	3.37	2.26	5.01	**<0.01**
Subjective health: Good (ref. Not good)	0.81	0.57	1.14	0.23
Exercise habit: Yes (ref. No)	1.24	0.88	1.73	0.22
Drinking habit: No (ref. Yes)	0.92	0.62	1.36	0.67
Smoking habit: No (ref. Yes)	1.38	0.89	2.15	0.15
Employment status: Employed (ref. Unemployed)	1.28	0.88	1.86	0.21
Educational background (ref. Junior high school)	1.37	1.12	1.68	**<0.01**
Radiation anxiety(For 1 point increase)	1.03	0.96	1.11	0.43
Discrimination and prejudice (For 1 point increase)	1.06	0.96	1.17	0.22

^a^Logistic regression analysis.

*p* < 0.05: statistically significant.

Abbreviations: CI, confidence interval; HL, health literacy; ref., reference.

Educational background: junior high school, lower high school/junior college, vocational school/university, graduate school; in ascending order.

## 4 Discussion

A previous study reported that health checkup participation was associated with individuals’ positive health beliefs (Okura et al., 2018). In the Health Belief Model, which has been widely used as a conceptual framework in health behavior research (Skinner et al., 2015), health behaviors have the potential to reduce risk of developing a disease, and individuals’ beliefs are linked to their health behaviors. This study focused on HL as an accelerator of “checkups” and “surveys” and aimed to examine associated factors of participation. The results of this study show no association between HL and participation in “checkups” and “surveys” by the residents of Fukushima Prefecture after the accident.

Previous studies that measured HL among the Japanese general population (Ishikawa et al., 2008, Ishikawa et al., 2016, Goto et al., 2019) have reported an average CCHL score ranging from 3.59 to 3.72. Since the average score for respondents in this study was 3.11 ± 0.81, it appears that the HL of Fukushima Prefecture residents is relatively lower than that of the other population in Japan. Large parts of Fukushima Prefecture is not an urban district compared to large cities such as Tokyo Metropolis, and the ratio of people having completed education up to colleges and universities was 10.3% in comparison with the whole of Japan (17.3%) (Estat, 2020). Previous studies have reported higher HL and education among citizens in urban areas compared with rural areas (Zahnd et al., 2009, Paasche-Orlow and Wolf, 2007). Lower score of HL in this study population might have reflected these socio-demographic characteristics. In addition, similar to the previous study, the proportion of employed respondents tended to be higher in the high HL group than in the low HL group; however, the difference was not statistically significant. A previous study reported that residents from the affected area of the nuclear accident might experience anxiety about radiation exposure resulting in a sharp decrease in the number of hospital staff after the accident (Ochi et al., 2016). Baker (2006) argued that HL depends on characteristics of the individual as well as the healthcare system. It is possible that many residents of Fukushima Prefecture feel anxious and find it difficult to understand the effects of radiation exposure on health, because they have no experience of accidental radiation exposure. Therefore, the circumstances surrounding the nuclear power plant accident might have lowered the HL of Fukushima Prefecture residents after the accident.

Contrary to our hypothesis, our results showed that HL was not associated with participation in either “checkups” or “surveys.” A previous study (Goto et al., 2018) also failed to identify an association between HL and participation in a health checkup in Japanese workers and suggested that health checkup participation is more affected by environmental factors, such as living arrangement, job demands, and having a primary doctor, than by HL. Also in the case of our study population, because they experienced the great disaster and the unexpected radiation accident, resulting in dramatic changes in their circumstances, the contribution of HL to “checkup” and “survey” participation might be relatively decreased. The complexities involved in understanding the effects of radiation on the health of residents might also help explain this contrary result. Furthermore, the items used to assess HL did not focus on information about radiation or its effects on health.

Although, we found no association with HL level and participation in “checkup” and “survey,” we did find that radiation anxiety was negatively associated with participation in “checkups.” Concerns about radiation risks were reportedly associated with psychological distress among evacuees of the Fukushima nuclear disaster (Suzuki et al., 2015). A previous study showed that non-participants of a comprehensive mass health examination had lower levels of subjective well-being and were more likely to be in a depressive state than participants (Suzuki et al., 2000). Thus, radiation anxiety could possibly be associated with worse mental health and, subsequently inhibit individuals from checking their general health. However, a reverse causality is also possible; people attending checkups had the opportunity to discuss with professionals, and were therefore less anxious, although causality was difficult to examine due to the nature of this cross-sectional study.

On the other hand, experiencing radiation anxiety led to a higher participation in “surveys” compared to participation for “checkups,” although this association was not significant. Radiation anxiety was reported to mediate immediate fear/anxiety after the accident leading to psychological distress (Fukasawa et al., 2017), which was the case in the respondents in our study. It is possible that they were motivated to participate in “surveys” to alleviate their accident-induced psychological distress.

Being employed was associated with participation in “checkups,” though not with “surveys.” In this study, some respondents work at companies and undergo health checkup with which their employer are obliged to provide them. The result of association between employment status and participation in “checkups” might have reflected this fact.

The increased risk of non-communicable diseases demonstrated the negative effect of the accident on the health of Fukushima Prefecture residents. These diseases appear to be the result of unhealthier lifestyles after the disaster. Residents in Minamisoma City who participated in the checkup reportedly experienced no cardiovascular health effects related to the nuclear accident (Toda et al., 2017). Since regular participation in “checkups” could promote good health, managing radiation anxiety might be significant for promoting health in residents after the accident.

As countermeasures, a previous study reported that instead of large-group and one-way communications, holding small group discussion to listen to participants’ concerns could help build rapport and might be effective in mitigating radiation anxiety (Murakami, et al. 2017). Another study suggested a gatekeeper training program for counselors to help residents cope with radiation anxiety (Orui, et al. 2020). Since this study could not confirm causal relationship between radiation anxiety and participation in “survey,” future study with a longitudinal design should be conducted to examine whether mitigating radiation anxiety will accelerate “checkup” participation.

This study has some limitations. First, the study design was cross-sectional; therefore, we could not determine any causal relationship between HL and participation, as well as associated factors. Second, the use of a self-reporting postal questionnaire might have caused low response rate, which led to low generalizability and inaccuracy of information as well as sampling bias. Furthermore, examination of generalizability was not possible because we could only collect name and address of non-responders, which made it impossible to compare characteristics between responders and non-responders. Third, data on lifestyle habits and participation in “checkups” before the disaster were not collected from the responders as such, we were unable to compare these variables before and after the disaster, or determine whether changes in lifestyle habits and/or “checkup” participation occurred due to the disaster. Fourth, although economic status represented as income was reported to be associated with participation in “checkups,” no supporting information was collected. Hence, the analysis did not adjust for economic status. A previous study regarding mechanism of effect of income on screening behavior suggested that income level affected the possession of private health insurance, which is related to higher participation in screening (Chang et al. 2015). Although health checkup and survey examined in this study were provided free of charge, lack of information on economic situation could affect the result on participation in “checkups.” Finally, the items used to collect information during the survey did not include any history or symptoms of any diseases. Hence, we were unable to analyze contributions of participation in “checkup” and “survey” on physical and mental health.

## 5 Conclusions

We observed that the HL of Fukushima Prefecture residents after the Fukushima Daiichi Nuclear Power Plant accident was lower than that reported previously for the Japanese. The residents’ HL level was not associated with their participation in “checkup” and “survey.” Instead, residing in the evacuation zone, being employed, less radiation anxiety was positively associated with participation in “checkups.” Residing in the evacuation zone at the time of the accident and a high level of education were positively associated with participation in “surveys.” Implementing measures to mitigate radiation anxiety might be beneficial in promoting participation in “checkups.”

## Funding

This study was supported by a grant from KAKENHI, 10.13039/501100001691Japan Society for the Promotion of Science (JSPS), as a Grant-in-Aid for Scientific Research (C) research [JSPS KAKENHI grant number 15K08810].

## CRediT authorship contribution statement

**Nobuaki Moriyama:** Formal analysis, Writing - original draft. **Chihiro Nakayama:** Conceptualization, Methodology, Formal analysis, Investigation, Resources, Data curation, Writing - review & editing, Visualization, Supervision, Project administration. **Masatsugu Orui:** Investigation, Writing - review & editing. **Yujiro Kuroda:** Investigation, Writing - review & editing. **Hajime Iwasa:** Writing - review & editing. **Teruko Horiuchi:** Writing - review & editing. **Takeo Nakayama:** Funding acquisition, Investigation, Writing - review & editing. **Minoru Sugita:** Funding acquisition, Investigation, Writing - review & editing. **Seiji Yasumura:** Conceptualization, Formal analysis, Resources, Data curation, Writing - review & editing, Visualization, Supervision, Project administration, Funding acquisition.

## Declaration of Competing Interest

The authors declare that they have no known competing financial interests or personal relationships that could have appeared to influence the work reported in this paper.
